# The Role of Short-Chain Fatty Acids in Acute Pancreatitis

**DOI:** 10.3390/molecules28134985

**Published:** 2023-06-25

**Authors:** Xiaxiao Yan, Jianing Li, Dong Wu

**Affiliations:** 1 Department of Gastroenterology, Peking Union Medical College Hospital, Chinese Academy of Medical Sciences and Peking Union Medical College, Beijing 100730, China; yanxiaxiao2@163.com (X.Y.); lijianing@263.net (J.L.); 2 Eight-Year Medical Doctor Program, Chinese Academy of Medical Sciences and Peking Union Medical College, Beijing 100730, China; 3 Clinical Epidemiology Unit, Peking Union Medical College Hospital, Chinese Academy of Medical Sciences and Peking Union Medical College, Beijing 100730, China

**Keywords:** acute pancreatitis, gut microbiota, short-chain fatty acids

## Abstract

Acute pancreatitis (AP) is a digestive emergency and can develop into a systematic illness. The role of the gut in the progression and deterioration of AP has drawn much attention from researchers, and areas of interest include dysbiosis of the intestinal flora, weakened intestinal barrier function, and bacterial and endotoxin translocation. Short-chain fatty acids (SCFAs), as one of the metabolites of gut microbiota, have been proven to be depleted in AP patients. SCFAs help restore gut homeostasis by rebuilding gut flora, stabilizing the intestinal epithelial barrier, and regulating inflammation. SCFAs can also suppress systematic inflammatory responses, improve the injured pancreas, and prevent and protect other organ dysfunctions. Based on multiple beneficial effects, increasing SCFAs is an essential idea of gut protective treatment in AP. Specific strategies include the direct use of butyrate or indirect supplementation through fiber, pre/pro/synbiotics, or fecal microbiota transplantation as a promising adjective therapy to enteral nutrition.

## 1. Introduction

Acute pancreatitis (AP) is a common digestive emergency and can develop into a systematic illness with a higher risk of death. According to the complications present, AP can be classified as mild (MAP), moderately severe (MSAP), or severe (SAP). About one-fifth of the cases will develop into SAP, which makes individuals prone to combined multi-organ failure (MOF) [1]. Organ dysfunction usually occurs in the early stage, and infection of pancreatic or peripancreatic necrotic tissue is the leading cause of death in the late stage. Furthermore, gut dysfunction plays a vital role in the deterioration of AP, which is associated with infectious complications and is described as the “motor of MOF” [2]. Recent studies have found that the progression of SAP is associated with early systemic inflammatory response syndrome (SIRS), dysbiosis of the intestinal flora, weakened intestinal barrier function, and bacterial and endotoxin translocation.

In AP patients, there are changes in intestinal flora diversity and composition. The increase of pathogenic bacteria and the reduction of probiotics have been detected, as well as decreased levels of short-chain fatty acids (SCFAs). SCFAs, mainly including acetate, propionate, and butyrate, are produced by microbial fermentation of undigested dietary carbohydrates in the intestine. There is a biological gradient for SCFAs from the gut lumen to the periphery [3]. As one of the intestinal flora’s metabolites, SCFAs directly provide energy for intestinal mucosal epithelial cells. The remaining part can be absorbed into the bloodstream to provide energy to other cells in the body [4,5]. SCFAs participate in glucose and lipid metabolism as substrates after transporting to hepatocytes and adipocytes and regulate appetite by interplaying with neurons [6,7]. SCFAs are also important signaling molecules involved in stabilizing the intestinal barrier and promoting intestinal immunity. Specifically, SCFAs protect the intestinal barrier by regulating the expression and distribution of tight junction proteins and promoting the secretion of mucin on the intestinal surface [8,9]. Moreover, SCFAs suppress the production of pro-inflammatory cytokines and promote immune cell recruitment, thus being regarded as a potential bioactive molecule for treating intestinal diseases [10,11]. There are two major functional pathways of SCFAs: the inhibition of histone deacetylase (HDAC) to exert epigenetic effects and the activation of G protein-coupled receptors (GPRs) to transfer signals [12]. Microbiota alterations and the decreased production of SCFAs are involved in the increase of intestinal permeability, leading to intestinal bacterial translocation, pancreatic tissue necrosis and infection, and even sepsis and MODS [13].

However, there is still a lack of specific evaluation and treatment methods for gut dysfunction [14]. No quantitative method exists to detect it clinically except for doctors’ observations. For example, the loss of intestinal peristalsis indicates impaired intestinal function. One of the most well-studied interventions is early enteral nutrition. It can mitigate mortality, organ failure, sepsis, and necrosis infection by replenishing caloric losses, increasing blood flow to preserve the bowel mucosa, and stimulating intestinal motility [15,16]. Other gut protection strategies include restoring intestinal dynamics, selective decontamination of the digestive tract, and regulating intestinal flora by probiotics. SCFAs will be another important intervention target for AP in the future. Here, we review the changes in microbiota and SCFAs in AP, their possible mechanisms, and the potential therapeutic value of SCFAs, collectively aiming to provide a new theoretical basis for intestinal protective therapy of AP.

## 2. Alteration of Gut Microecology in AP

The role of dysbiosis in pancreatic diseases has attracted much attention with the rapid evolvement of microbiota research. Compared with healthy controls, AP is accompanied by intestinal microecological dysbiosis, although in the early stages of the disease course. Multiple studies found that the diversity of intestinal flora decreased either in AP patients or mice models (Table 1). The increased pathogenic bacteria and decreased probiotics are prominent features. At the phylum level, increased Bacteroidetes and Proteobacteria were found in AP patients with fewer Firmicutes and Actinobacteria. At the genus level, the main characteristic alterations were increased *Enterobacteriaceae*, *Enterococcus*, and *Escherichia-Shigella*, with decreased *Bifidobacterium* [17,18]. The main secretory products of *Bifidobacterium* are acetic acid and lactic acid, which can lower the intestinal pH and inhibit the growth of harmful intestinal bacteria. Reduced *Bifidobacterium* are also recognized cellulose degraders that promote the fermentation of dietary fiber and produce SCFAs. Stratified analysis of AP patients found that the microbiota alteration differed with the disease severity. Among these alterations, the decrease of SCFA-producing bacteria affected the integrity of the intestinal barrier and worsened the severity of AP. Zhu et al. reported that SAP was characterized by reduced commensal bacteria, such as *Bacteroides*, *Alloprevotella*, and *Blautia*. Their linear discrimination analysis coupled with effect size (LEfSe) analysis revealed a significant increase of *Acinetobacter*, *Stenotrophomonas*, and *Geobacillus* and a substantial reduction of *Bacteroides*, *Alloprevotella*, *Blautia*, and *Gemella* in SAP than mild acute pancreatitis (MAP) and moderately severe acute pancreatitis (MSAP) [18]. Previous studies have shown that these reduced organisms could facilitate fermentation and produce SCFAs [19,20,21,22]. Yu et al. found more *Bacteroides*, *Escherichis-Shigella*, and *Enterococcus* in MAP, MSAP, and SAP, respectively. Moreover, *Eubacterium hallii*, one of the butyrate-producing bacteria, was the most decreased strain in MASP and SAP patients [23].

Recently, investigations of microbial composition have extended to the pancreas, which was once thought to be a sterile organ. Several studies supported the fact that microorganisms inhabit the pancreas in a nonpathological state, namely inherent pancreatic microbiota [24,25]. However, there has not been a specific definition of normal pancreatic microbiota based on the limited research. Potential sources and routes of pancreatic bacteria include the esophagus, stomach, duodenum, or biliary tract microbiota via the pancreatic duct or translocation from the lower gastrointestinal tract through the portal circulation or mesenteric lymph nodes [14]. Pancreatic infection is a significant cause of complications and death in patients with acute necrotizing pancreatitis (ANP). About one-third of the patients with pancreatic necrosis will develop infectious necrosis [1], but it is more common later in the clinical course. Most infections are single intestinal microbial infections, such as *Escherichia coli*, *Pseudomonas*, *Klebsiella*, and *Enterococcus*, indicating the translocation of gut bacteria [26,27]. An ANP mice model study evaluated the spatial (lumen versus mucosa) and regional composition and function of the microbiota from the duodenum, ileum, caecum, colon, pancreas, and blood using 16S rRNA gene amplicon sequencing. Results showed that the distal gut microbiota was significantly impacted, and the duodenal microbiota might also play a role in bacterial translation and secondary infections [28].

**Table 1:** Gut microbiota dysbiosis in acute pancreatitis compared with healthy controls.

Studies	Subjects	Sample	Phylum	Genus	Species
Tian 2015 [29]	76 AP and 32 HC	Fecal		*Enterobacteriaceae* ↑ *Enterococcus* ↑ *Bifidobacterium* ↓	
Zhang 2018 [17]	45 AP and 44 HC	Fecal	Bacteroidetes ↑ Proteobacteria ↑ Firmicutes ↓ Actinobacteria ↓		
Zhu 2019 [18]	130 AP and 35 HC	Fecal	Proteobacteria ↑ Bacteroidetes ↓	*Escherichia/Shigella* ↑ *Enterococcus* ↑ An unknown genus in family of *Enterobacteriaceae* ↑ *Prevotella_9* ↓ *Faecalibacterium* ↓ *Blautia* ↓ *Lachnospiraceae* ↓ *Bifidobacterium* ↓	
Yu 2020 [23]	60 AP and 20 HC	Rectal swab		*Finegoldia* ↑ *Anaerococcus* ↑ *Enterococcus* ↑ *Eubacterium hallii* ↓	*Blautia* ↓ *Finegoldia* ↑
van den Berg 2021 [30]	35 AP and 15 HC	Fecal	Proteobacteria ↑	*Escherichia/Shigella* ↑ *Streptococcus* ↑ Butyrate producers ↓ ^1^	

AP, acute pancreatitis; HC, health controls. The arrows indicate the alteration of gut microbiota in AP compared with healthy controls. ^1^ A panel of butyrate producers based on genus taxonomy was constructed based on butyrate-producing taxa (*Alistipes*, *Anaerostipes*, *Butyricicoccus*, *Butyricimonas*, *Butyrivibrio-010*, *Coprococcus_1*, *Coprococcus_2*, *Coprococcus_3*, *Eubacterium*, *Faecalibacterium*, *Flavonifractor*, *Odoribacter*, *Oscillibacter*, *Pseudoflavonifractor*, *Roseburia*, *Ruminococcus_2*, *Subdoligranulum*).

## 3. Function of SCFAs in AP

### 3.1. Mitigation of Intestinal Injury

Gut barrier dysfunction is present in three of five patients with AP, which is associated with poor clinical outcomes [31]. Considering the site of SCFAs production, most studies on the mechanism of SCFAs focused on intestinal homeostasis [32]. Intestinal homeostasis is an organic and dynamic balancing state involving the gut microbiota, the intestinal epithelial barrier, and the mucosal immune barrier. The protective role of SCFAs can also be summarized in these three aspects. SCFAs can rebuild the disordered intestinal flora. After butyrate supplementation, the abundance of SCFA-producing *Alloprevotella* and *Muribaculaceae* increased [33,34]. SCFAs also act directly on the intestinal epithelium to protect the integrity of the intestinal barrier, which can be observed at the histological level. Moreover, SCFAs are a critical carbon source for colonic enterocytes [35]. SAP rats in the butyrate treatment group showed mitigated mucosa lesions and decreased epithelial apoptosis. Butyrate protected the intestinal barrier by upregulating tight junction proteins, such as zonula occludens-1 (ZO-1), claudin, and occludin [33,34]. In addition to the epithelial barrier repairment, butyrate also well-restored mucin-secreting goblet cells, thus protecting the damaged mucous membrane [33]. For the immunity barrier, pre-treatment with sodium butyrate ameliorated intestinal inflammation and injury by reducing intestinal pro-inflammatory cytokines, including tumor necrosis factor (TNF)-α and interleukin (IL)-6. Butyrate also increased the expression of Foxp3 at both mRNA and protein levels, detected in immunofluorescence staining and flow cytometry analysis. These results supported the elevated percentage of regulatory T cells (CD4+, CD25+, Foxp3+), which could maintain intestinal homeostasis by preventing inappropriate innate and adaptive immune responses [36,37,38].

SAP patients can develop intraabdominal hypertension (IAH), which can progress to abdominal compartment syndrome (ACS) with a high mortality rate of 66.7%. These severe abdominal complications may correlate with pancreatic necrosis and intra-abdominal infection, likely resulting from bacterial translocation [39,40,41]. *Clostridium butyricum* is an anaerobic bacterium that can ferment dietary fibers to produce SCFAs. SAP + IAH rats who received oral *C. butyricum* or butyrate had reduced pathological severity scores of intestinal injury and plasma levels of inflammatory markers. Compared with the nontreated group, the expression of ZO-1, claudin-1, and occludin increased, and claudin-2, matrix metallopeptidases 9 (MMP9), and TNF-a lowered in the treatment group, indicating repairment of the intestinal mucosal barrier. The treatment also rebuilt the intestinal flora, significantly increasing richness and diversity, growing probiotics (*Lactobacillus*, *Coprococcus*, and *Allobaculum*), and decreasing pathogenic species (*Bacteroides*, *Escherichia*, *Helicobacter*, and *Desulfovibrio*) [42]. These multiple reversed pathological responses suggested butyrate supplementation as a promising therapeutic strategy to restore intestinal function. Another study further confirmed the protective effect of *C. butyricum* or butyrate via downregulating MMP9 expression [43]. MMP9 was upregulated in intestinal tissues of the SAP model according to existing studies [44,45], and is one of the members of MMPs that can degrade and remodel extracellular matrix. MMPs are also involved in the inflammation process and intestinal barrier injury. For example, MMPs can increase endothelial cell permeability by disrupting tight junction proteins [46]. Additionally, Kocael et al. reported that MMP9 overexpression also facilitated the loss of intestinal villous in the mesenteric ischemia-reperfusion injury model [44]. Therefore, MMP9 is a vital molecule mediating intestinal injury and a potential target of SCFA supplementation.

### 3.2. Reduction of Pancreas Injury

There has been evidence for the direct interaction between the pancreas and SCFAs and the existence of the gut–pancreatic axis [47]. Cathelicidin-related antimicrobial peptide (CRAMP) production by insulin-secreting beta-cells is controlled by SCFAs produced by the gut microbiota. However, local functions of SCFAs in the pancreatic tissue of AP are limitedly studied. In the AP mice model, butyrate mitigated the severity of AP in multiple ways, reflected in both the pancreas and the gut. A study provided new insights into tissue-specific mechanisms of butyrate. Pre-treatment with sodium butyrate decreased the infiltration of macrophages and neutrophils in the pancreas and reduced levels of intestinal pro-inflammation cytokines. Sodium butyrate acted as an HDAC1 inhibitor in the pancreas or as a GPR109A agonist in the colon to suppress the activation of NLRP3 inflammasome [48]. Lei et al. also reported that, in the heparanase-exacerbated AP model, the supplementation of Parabacteroides or sodium acetate could reduce neutrophils in blood and infiltration in the pancreas [49]. Similarly, in another study, sodium butyrate supplementation significantly reduced the proportion of neutrophils, macrophages, and M2-type macrophages in the pancreatic tissue from AP mice and inhibited IL-1b, CXCL1, and TNF-a levels [34].

### 3.3. Prevention and Protection of Other Organ Dysfunctions

In the early phase of AP, inflammation of the pancreas activates cytokine cascades, which are clinically manifested as SIRS [50]. Avoidance of SIRS or timely termination of SIRS is the key to early control of AP. Fecal concentrations of butyrate, propionate, and acetate in patients with severe SIRS on admission decreased significantly compared with those in healthy volunteers. They remained low throughout the six weeks of intensive care unit (ICU) stay. In patients with gastrointestinal complications, including enteritis and dysmotility, the level of SCFAs was even lower [51]. Zhang et al. found that sodium butyrate treatment could inhibit the nuclear factor-κB (NF-κB) signaling pathway and lower the expression of High-mobility group box-1 (HMGB1), which is a late cytokine mediator stimulating the release of pro-inflammatory cytokines. The SAP model had reduced pathological lesions; reduced serum levels of HMGB1, TNF-a, and IL-6; as well as diminished HMGB1 mRNA levels and NF-κB activity [52]. Another study that used the SAP model also evaluated the plasma levels of several markers. The administration of *Clostridium butyricum* or butyrate reduced pro-inflammatory cytokines, including TNF-a, IL-6, IL-1β, and IL-12 [42]. What is more, SCFAs mitigated the inflammation in the lipopolysaccharide (LPS)-induced septic shock model by upregulating the anti-inflammatory cytokine IL-10 [53].

Patients with persistent SIRS are at risk for one or more organ failures, the leading cause of early death. Three organ systems should be assessed to define organ failure, including respiratory, cardiovascular, and renal systems. Organ failure may be transient with remission within 48 h in MSAP or persistent for more than 48 h in SAP [50]. SCFAs prevent or protect against organ failures by restoring the intestinal barrier and suppressing systematic inflammatory responses. Some changes mediated by SCFAs in specifically targeted organs, such as the lung and kidney, are also observed.

One-third of patients presenting with severe AP develop acute lung injury (ALI) and acute respiratory distress syndrome (ARDS). The primary manifestation is hypoxemia, which can even develop into acute respiratory distress syndrome (ARDS). The lung injury is characterized by increased pulmonary microvasculature permeability and subsequent protein-rich exudate leakage into the alveolar spaces, forming the hyaline membrane [54,55]. The concept of the gut–lung axis has been proposed based on the bidirectional crosstalk between these two organs [56]. SCFAs provide one of these crosstalk pathways. Human lung tissue contained variable acetate and propionate levels, likely originating from the gut and transiting to the lung. SCFA receptors, namely free fatty acid receptor (FFAR) 2 and FFAR3, were expressed in vitro in alveolar macrophages (AM) and alveolar type 2 epithelial (AT2) cells, and exposure to LPS regulated this expression. This finding supported the direct effects of SCFAs on the lung [57]. Specifically, gut microbiota-produced LPS and SCFAs could strongly influence the course of lung injury and infections [58,59]. SCFAs significantly protected animals from LPS-induced ALI, as evidenced by suppressed HMGB1 release and NF-κB activation, decreased production of pro-inflammatory cytokines and reactive oxygen species, declined immune cell counting, and alleviated LPS-induced microvascular permeability and lung histological damage [60,61,62]. In the hypoxic model, butyrate treatment decreased the accumulation of alveolar and interstitial lung macrophages, prevented hypoxia-induced pulmonary vascular edema and vascular leakage, and upregulated the expression of tight junctions in lung microvascular endothelial cells [63]. Tian et al. similarly found that enrichment of propionate-producing gut bacteria (especially *Lachnospiraceae*) was related to reduced lung inflammation following lung ischemia-reperfusion injury in vivo [58]. Compared with AP patients without ARDS, AP with ARDS had higher abundances of the Proteobacteria phylum, the *Enterobacteriaceae* family, *Escherichia-Shigella*, and the *Klebsiella pneumoniae* genus but lower abundances of the *Bifidobacterium* genus [64]. Thus, gut microbiota and SCFAs may play essential roles in pancreatitis-associated lung injury through the above mechanisms, although no studies used the AP model.

Acute kidney injury (AKI) is another frequent complication of SAP. A comprehensive, retrospective, observational study reported an overall AKI prevalence of 7.9% among hospitalized patients with AP [65]. The pathogenesis may include increased vascular permeability, hypovolemia, inflammation, vasoconstriction, intravascular coagulation, and direct nephrotoxic effects [66]. Predictors for a higher likelihood of AKI include higher age, exhibiting biological male sex, a more significant number of co-morbidities, and electrolyte imbalance [65]. Andrade-Oliveira et al. observed that therapy with the three main SCFAs (acetate, propionate, and butyrate) improved renal dysfunction. In specific, SCFAs treatment reduced pro-inflammatory cytokines and chemokines in kidney tissue and serum, with low levels of toll-like receptor 4 (TLR4) mRNA and lesser activation of the NF-κB pathway. SCFA treatment also diminished apoptotic cells in kidney tissue, but increased the proliferation of kidney epithelial cells, thus promoting the restoration of injured tissue. Mice treated with acetate-producing bacteria also achieved better outcomes after AKI, having increased acetate levels in feces and plasma, low serum levels of creatinine and urea, and low serum levels of cytokines and chemokines [67]. Another study showed that a high-fiber diet had similar protective effects for AKI [68]. Zhang et al. evaluated other organs in the SAP model after sodium butyrate treatment and found alleviated liver and renal tissue histological injuries and improved hepatic and renal function reflected in decreased alanine aminotransferase and creatine levels [52].

**Figure 1:**
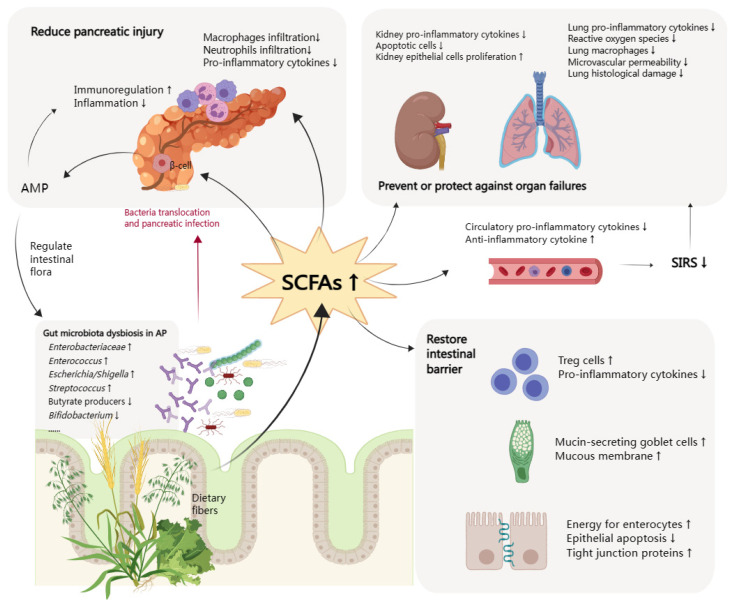
Function of short chain fatty acids in acute pancreatitis. SCFAs, short chain fatty acids; AMP, antimicrobial peptide; SIRS, systemic inflammatory response syndrome; AP, acute pancreatitis; Treg cells, regulatory T cells. The arrows (↑ and ↓) indicate the alternation of gut microbiota in AP or histopathological changes after increasing SCFAs.

The late phase of AP is characterized by the persistence of systematic signs of inflammation or local complications, with an increased risk of infection. Up to 20% of AP patients develop extra-pancreatic infections, such as bloodstream infections, pneumonia, and urinary tract infections [69]. In a meta-analysis of studies performed in the ICU, there was a significantly lower risk of infection in the patients who received early enteral nutrition. Infectious complications occurred in 19% of the early nutritional group compared to 41% in the delayed group [70]. Considering that SCFAs can protect the intestinal barrier and prevent bacteria translocation, SCFAs are a good choice to reduce the incidence of systemic infectious complications of AP as a supplement to early enteral nutrition. The above-mentioned possible mechanisms of SCFAs in AP are summarized in Figure 1.

## 4. Treatment Potential of SCFAs in AP

### 4.1. Dietary Fiber Supplementation

Critically ill SAP patients treated with enteral nutrition and broad-spectrum antibiotics often present with diarrhea, indicating intestinal dysfunction. Compared with healthy volunteers, fecal microbiota mass and SCFAs were significantly lower in critically ill SAP patients, indicating gut dysbiosis and suppression of colonic fermentation. After 2–5 week fiber supplementation, there was a six-fold increase in fecal SCFAs and microbial counts of specific butyrate producers, with the resolution of diarrhea [71,72]. In a prospective double-blind randomized controlled trial (RCT) conducted by Karakan et al., nasojejunal enteral nutrition with prebiotic fiber supplementation in SAP improved hospital stay, duration of nutrition therapy, acute phase response, and overall complications compared to standard enteral nutrition therapy [73]. Therefore, fiber supplementation may preserve gut function in critically ill patients through increased SCFAs. It is an easy and safe improvement of standard enteral nutrition. However, the efficacy of fiber supplementation may be related to the abundance of SCFA-producing bacteria and intestinal motility.

### 4.2. Probiotics, Prebiotics, and Synbiotics

Due to the controversial evidence from clinical trials, pre/pro/synbiotics for AP have not been positively accepted and recommended in guidelines. The main conclusions of research on probiotics have undergone several changes in the last 20 years (Table 2). An early small-scale RCT reported that specific lactobacillus and fiber supplements effectively reduced pancreatic sepsis and the number of surgical interventions in AP [74]. Another RCT suggested that early nasojejunal feeding with synbiotics (*Lactobacilli* preparations and bioactive fibers) may prevent organ dysfunctions in the late phase of SAP [75]. However, a Dutch study showed that for patients with predicted SAP, probiotic prophylaxis with a combination of probiotic strains (*Lactobacillus acidophilus*, *Lactobacillus casei*, *Lactobacillus salivarius*, *Lactococcus lactis*, *Bifidobacterium bifidum*, and *Bifidobacterium lactis*) did not reduce the risk of infectious complications and was associated with an increased risk of mortality [76]. This study provided unexpected and exactly opposite results compared to previous RCTs. This unfavorable result might be related to a high prevalence of bowel ischemia in the treatment group. The authors provided a proposed mechanism that a high dose of bacteria combined with enteral nutrition increased oxygen demand and/or local mucosal effect. What is more, the higher AP severity, more types, higher dose and longer duration of probiotic organisms, and more aggressive hypercaloric enteral feeding compared with previous studies might also be influencing factors [77,78,79,80]. These conflicting results remind us that the optimized selection, dose, timing, delivery, and patient population of probiotics use are still pending further investigation. Subsequent trials demonstrated no increase in mortality or morbidity, with fewer infectious complications, multi-organ dysfunction, and decreased pro-inflammatory markers [81,82]. According to the pooled results of a systematic review including nine RCTs, pre/pro/synbiotics reduced the risk of organ failure and length of hospital stay in patients with SAP. Still, no difference was observed for SIRS, infected pancreatic necrosis, surgical intervention, septic morbidity, and mortality [83].

Prebiotic lactulose is a potent choice in treating AP patients suffering from gut failure. A recent prospective randomized trial compared the efficacy of lactulose and rhubarb in MSAP patients with intestinal dysfunction [84]. Lactulose had better performance in decreasing the serum levels of cytokines and gut permeability index, enriching the potential beneficial genus *Bifidobacterium* and inhibiting *Escherichia-Shigella*. Of note, the level of SCFAs remarkably increased after treatment, with a higher amount in the lactulose group than in the rhubarb group. In another study, Rohith et al. evaluated the efficacy of synbiotics (containing *Streptococcus faecalis* T-110, *Clostridium butyricum* TO-A, *Bacillus mesentricus* TO-A, and *Lactobacillus sporogenes*) in MSAP and SAP [85]. The results indicated that the value of synbiotics was limited, which only lowered bacteremia and length of hospitalization, but septic complications and mortality were not significantly different. Further studies must address some crucial questions, including the selection of potential beneficiary patient populations, the formulation of pre/pro/synbiotics, the optimal administration timing, and the treatment duration. Safety in particular requires attention when using live bacteria.

**Table 2:** Use of probiotics, prebiotics, and synbiotics in AP.

Studies	Subjects	Pre/Pro/Synbiotics	Main Effect of Treatment Group
Oláh 2002 [74]	45 AP	Live *L. plantarum* 299, together with a substrate of oat fiber	Pancreatic sepsis ↓ Number of surgical interventions ↓
Oláh 2007 [75]	62 SAP	Four different *lactobacilli* preparations and prebiotics containing four bioactive fibers (inulin, beta-glucan, resistant starch, and pectin)	Incidence SIRS and MOF ↓ Rate of late (over 48 h) organ failure ↓
Karakan 2007 [73]	30 SAP	Standard enteral nutrition with soluble and insoluble fibers	Hospital stay ↓ APACHE II, CRP, and CT store normalization duration ↓ Overall complications ↓
Besselink 2008 [76]	298 predicted SAP	*Lactobacillus acidophilus*, *Lactobacillus casei*, *Lactobacillus salivarius*, *Lactococcus lactis*, *Bifidobacterium bifidum*, and *Bifidobacterium lactis*	Risk of mortality ↑
Lata 2010 [86]	22 AP	*B. bifidum*, *B. infantis*, *L. acidophilus*, *L. casei*, *L. salivarius*, *L. lactis*	Endotoxin levels ↓
Sharma 2011 [82]	50 AP	*Lactobacillus acidophilus*, *Bifidobacterium longus*, *Bifidobacterium bifidum*, and *Bifidobacterium infantalis* with fructo-oligosaccharide	CRP and immunoglobulins ↓
Plaudis 2012 [87]	90 SAP	Synbiotic 2000 Forte	Infection rate (pancreatic and peripancreatic necrosis) ↓ Rate of surgical interventions ↓ ICU and hospital stay ↓ Mortality rate ↓
Cui 2013 [81]	70 SAP	*Bifidobacterium*	Pro-inflammatory cytokines ↓ Earlier restoration of gastrointestinal function Complications ↓ Hospital day ↓
Wang 2013 [88]	183 SAP	Live *Bacillus subtilis* and *Enterococcus faecium*	Percentage of pancreatic sepsis and MODS ↓ Mortality rate ↓ Pro-inflammatory cytokines and APACHE II scores ↓ Plasma concentration of IL-10 ↑
Zhu 2014 [89]	39 SAP	*C. Butyricum*	Rate of intestinal ischemia and necrosis ↑
Li 2014 [90]	80 SAP	*Bifidobacterium*	Pro-inflammatory cytokines levels ↓ CRP and LDH levels ↓ Mortality and incidence of complications ↓ Duration of hospitalization ↓
Wu 2017 [91]	120 SAP	Live *B. bifidus*, *B. acidophilus*, *E. faecalis*, and *B. cereus*	Incidence of infection MODS ↓ Duration of abdomen pain and hospitalization ↓
Fang 2018 [92]	68 SAP	Live *Bifidobacterium*, *Lactobacillus*, and *Enterococcus*	Relieved clinical symptoms Hospitalization time ↓ Serum inflammatory cytokine levels ↓
Wang 2023 [84]	73 MSAP	Lactulose	Serum levels of cytokines ↓ Gut permeability index ↓ Bifidobacterium ↑ Level of SCFAs ↑
Rohith 2023 [85]	86 MSAP or SAP	Synbiotics containing *Streptococcus faecalis* T-110, *Clostridium butyricum* TO-A, *Bacillus mesentricus* TO-A, and *Lactobacillus sporogenes*	Total leucocyte and neutrophil counts ↓ Length of hospitalization ↓

AP, acute pancreatitis; MSAP, moderately severe acute pancreatitis; SAP, severe acute pancreatitis; SIRS, systemic inflammatory response syndrome; MOF, multi-organ failure; MODS, multiple organ dysfunction syndrome; APACHE II, Acute Physiology and Chronic Health Evaluation II; IL, interleukin; CRP, C-reactive protein; ICU, intensive care unit; CT, computed tomography; LDH, lactate dehydrogenase; SCFAs, short chain fatty acids. The arrows indicate the changes of main outcomes after the treatment of probiotics, prebiotics, or synbiotics.

### 4.3. Direct Supplementation of SCFAs

In 2019, the International Scientific Association for Probiotics and Prebiotics (ISAPP) proposed that a postbiotic is a “preparation of inanimate microorganisms and/or their components that confers a health benefit on the host”. Postbiotics are deliberately inactivated microbial cells with or without metabolites or cell components contributing to demonstrated health benefits [93]. SCFAs are one group of these beneficial metabolites. According to the mechanisms discussed above, SCFAs benefit AP on different levels, including regulating local and systematic inflammation, restoring intestinal barrier function, and reversing other organ dysfunctions. A research team from the University of Amsterdam used a mouse model of ANP fed with the Western diet, which contained 60% polyunsaturated fatty acids and no soluble fibers. The Western diet caused a bloom of *Escherichia/Shigella* and increased mortality and systemic infection in ANP mice. There was also a significant decrease in butyrate, amino acids, and carbohydrates. Collectively, these results confirmed that the Western diet is involved in AP pathogenesis. For therapeutic strategies, both oral and intraperitoneal butyrate reduced mortality and *E. coli* dissemination and reversed the microbiota alterations [30]. Although SCFAs have been subjected to clinical trials in humans with some encouraging results, including ulcerative colitis, radiation proctosigmoiditis, and visceral hypersensitivity, there have not been clinical trials about the administration of SCFAs in AP patients, and the current findings were all from animal models [94,95,96,97,98,99,100,101,102,103,104]. Direct SCFAs supplementation in enteral nutrition for AP treatment is essential for future clinical studies. It is expected to be safer than probiotics, which might cause bacteremia. However, SCFAs used as purified substances and not as a component of an inactivated microbial preparation would not be considered postbiotics.

### 4.4. Fecal Microbiota Transplantation

Fecal microbiota transplantation (FMT) is a direct approach to restoring the intestinal environment. It has been recommended to treat *Clostridium difficile* infection (CDI) [105]. The US Food and Drug Administration (FDA) issued a safety alert for using FMT after reports of serious adverse effects, including death due to infections with multidrug-resistant bacteria [106,107]. Yang et al. reported a case of MSAP complicated with severe CDI who suffered from diarrhea during his AP course. This 51-year-old man was treated by FMT as a first-line therapy. During the treatment, no adverse events were reported. Diarrhea resolved within five days after FMT. The patient remained asymptomatic, and the follow-up colonoscopy performed 40 days after discharge showed a complete recovery. However, the effect of FMT on AP was not evaluated and reported [108]. There are only some FMT-related studies conducted in AP mice. The bacteria translocation and mortality rate were significantly increased in pancreatitis mice that received FMT from healthy mice [30]. Another study reported that normobiotic FMT alleviated AP-induced gut microbiota dysbiosis and lessened the severity of AP, including mitochondrial dysfunction, oxidative damage, and inflammation. Gut microbiota-derived nicotinamide mononucleotide may play an essential role in this process [109]. According to these limited but controversial results from animal experiments, the effects and safety of FMT in AP treatment undoubtedly need further research.

## 5. Conclusions

Mucosal barrier protection is the common pathophysiological basis of almost all gastrointestinal diseases. Although the inflammation in AP starts in the pancreas, the gut plays an amplifier role in the disease course, leading to aggravated, even uncontrolled, inflammatory responses. Therefore, in treating AP, maintaining intact intestinal function is a significant part of controlling inflammation. SCFAs are a group of gut microbiota metabolites that could help rebuild the intestinal epithelial barrier and suppress inflammatory responses, thus maintaining a healthy intestinal environment. In addition to the intestine as the most important first step, SCFAs also reduce systematic inflammation and protect other organ functions, such as lung and kidney, which are frequently involved in AP. Therefore, supplementation of SCFAs directly or indirectly is a promising therapeutic approach, although existing research results are limited and controversial. More well designed clinical trials are needed for the comprehensive and individualized application of SCFAs, and safety is noteworthy, considering the complexity and aggressiveness of AP.

## Data Availability

Not applicable.
